# Use of miniaturized extracorporeal circulation for implanting left ventricular assist devices in high-risk end-stage heart failure patients

**DOI:** 10.1186/1749-8090-8-S1-O144

**Published:** 2013-09-11

**Authors:** P Antonitsis, H Argiriadou, P Tossios, A Deliopoulos, S Westaby, K Anastasiadis

**Affiliations:** 1 Department of Cardiothoracic Surgery, AHEPA University Hospital, Thessaloniki, Greece; 2 Department on Anaesthesia and Intensive Care, AHEPA University Hospital, Thessaloniki, Greece; 3 Department of Cardiothoracic Surgery, John Radcliffe Hospital, Oxford, UK

## Background

Miniaturized extracorporeal circulation (MECC) has been implemented in clinical practice for the last decade with superior results regarding postoperative morbidity and mortality mainly in coronary procedures. We challenged this technique in high-risk patients with end-stage heart failure undergoing left ventricular assist device (LVAD) implantation.

## Methods

Four patients with end-stage ischemic heart failure were included in this study. All patients were inotrope dependent (INTERMACS class 2) and their preoperative evaluation considered them to be high-risk for developing postoperative right ventricular failure. Hence, optimization of right ventricular function was performed preoperatively following a protocol based on intermittent administration of doses of levosimendan. Two Jarvik 2000 and two HeartWare LVADs were implanted on beating heart using MECC system. Surgical strategy included retrograde autologous priming of the circuit and avoidance of administering cardioplegia.

## Results

All procedures were uneventful and patients were weaned-off MECC on the LVAD smoothly. There was no need for nitric oxide administration either intraoperatively or in the ICU. No blood or blood product was transfused intraoperatively while minimal blood transfusion was required postoperatively. Renal and hepatic laboratory parameters were maintained within normal range. All patients were discharged from the ICU in a median of 4 days without need for inotropic support.

## Conclusions

This is the first reported series on the use of MECC in high-risk patients undergoing LVAD implantation. MECC provides haemodynamic stability throughout the procedure, while it minimizes haemodilution and preserves right ventricular function, which is of utmost importance. We believe that MECC use without ischemic time for the myocardium should be the standard operative technique for LVAD implantation, especially in high-risk patients.

